# Toward reliable population density estimates of partially marked populations using spatially explicit mark–resight methods

**DOI:** 10.1002/ece3.4907

**Published:** 2019-01-24

**Authors:** Andrew Carter, Joanne M. Potts, David A. Roshier

**Affiliations:** ^1^ Australian Wildlife Conservancy Subiaco East Western Australia Australia; ^2^ Institute for Land, Water and Society Charles Sturt University Albury New South Wales Australia; ^3^ The Analytical Edge Pty Ltd Blackmans Bay Tasmania Australia; ^4^ Centre for Ecosystem Science University of New South Wales Sydney New South Wales Australia

**Keywords:** camera trap, capture–recapture, fox, maximum likelihood, mesopredator, survey design, *Vulpes vulpes*

## Abstract

1. Camera traps are used increasingly to estimate population density for elusive and difficult to observe species. A standard practice for mammalian surveys is to place cameras on roads, trails, and paths to maximize detections and/or increase efficiency in the field. However, for many species it is unclear whether track‐based camera surveys provide reliable estimates of population density.
2. Understanding how the spatial arrangement of camera traps affects population density estimates is of key interest to contemporary conservationists and managers given the rapid increase in camera‐based wildlife surveys.
3. We evaluated the effect of camera‐trap placement, using several survey designs, on density estimates of a widespread mesopredator, the red fox *Vulpes vulpes*, over a two‐year period in a semi‐arid conservation reserve in south‐eastern Australia. Further, we used the certainty in the identity and whereabouts of individuals (via GPS collars) to assess how resighting rates of marked foxes affect density estimates using maximum likelihood spatially explicit mark–resight methods.
4. Fox detection rates were much higher at cameras placed on tracks compared with off‐track cameras, yet in the majority of sessions, camera placement had relatively little effect on point estimates of density. However, for each survey design, the precision of density estimates varied considerably across sessions, influenced heavily by the absolute number of marked foxes detected, the number of times marked foxes was resighted, and the number of detection events of unmarked foxes.
5. Our research demonstrates that the precision of population density estimates using spatially explicit mark–resight models is sensitive to resighting rates of identifiable individuals. Nonetheless, camera surveys based either on‐ or off‐track can provide reliable estimates of population density using spatially explicit mark–resight models. This underscores the importance of incorporating information on the spatial behavior of the subject species when planning camera‐trap surveys.

## 1 INTRODUCTION

The reliable estimation of population densities is a key element of any conservation management strategy, whether the species of interest is a conservation asset or threat (Soisalo & Cavalcanti, 2006). Many species are elusive and difficult to observe due to behaviors such as nocturnality or because they occur at low densities. In such circumstances, camera traps are used increasingly to detect species presence and estimate density (Ordeñana et al., 2010; Sollmann, Gardner, Parsons et al., 2013; Towerton, Penman, Kavanagh, & Dickman, 2011). The most reliable methods for estimating population density use models that incorporate spatial attributes (geographic coordinates) of both the camera traps and where animals are recorded. Selecting the most appropriate method depends on whether animals are identifiable individually. If no individuals are identifiable, options include random encounter modeling (Rowcliffe, Field, Turvey, & Carbone, 2008), spatial presence–absence (Ramsey, Caley, & Robley, 2015), and *N*‐Mixture models (Jiménez et al., 2017; Royle, 2004). If a proportion of the population is identified individually, spatially explicit mark–resight (SEMR) methods are suitable (Rich et al., 2014; Sollmann, Gardner, Chandler et al., 2013; Sollmann, Gardner, Parsons et al., 2013), while spatially explicit capture–recapture (SECR) is appropriate if all animals recorded are identifiable (Alexander, Gopalaswamy, Shi, & Riordan, 2015; Bahaa‐el‐din et al., 2016; Borchers & Efford, 2008; Hearn et al., 2016; Royle & Young, 2008). Models are also available that combine data collection methods, for example, when animals cannot be identified uniquely (i.e., providing an encounter rate) but telemetry movement data are available for a portion of the population (e.g., Potts, Buckland, Thomas, & Savage, 2012; White & Shenk, 2001).

Regardless of the analytical method selected, the reliability of density estimates depends on appropriate survey design—an issue that has generally been undervalued in camera‐trap studies (Meek, Ballard, & Fleming, 2015). Camera placement is a fundamental design decision that affects detection probability, and some designs will introduce biases into density estimates. The only truly unbiased design is to position all cameras randomly within the study area, although this strategy can reduce detectability for some species as frequently used locations are not targeted, typically increasing the uncertainty of density estimates. Nonetheless, placing cameras on roads, trails, and paths is standard practice for surveying carnivores (e.g., Anile, Ragni, Randi, Mattucci, & Rovero, 2014; Meek, Ballard, Fleming, & Falzon, 2016; Sollmann et al., 2011); either for logistic reasons (e.g., to more efficiently survey large areas) or to maximize detections of elusive species that frequently utilize trails (Karanth & Nichols, 1998; Sollmann et al., 2011). Here, we examine the trade‐offs between camera placement and density estimation for a common mesopredator with non‐distinctive pelage, the red fox *Vulpes vulpes*, in a semi‐arid conservation reserve in south‐eastern Australia.

In Australia, introduced mesopredators (foxes and feral cats *Felis catus*) have driven the decline or extinction of one‐third of the island continent's endemic terrestrial mammals (Doherty, Glen, Nimmo, Ritchie, & Dickman, 2016; Fleming et al., 2014; Woinarski, Burbidge, & Harrison, 2015). Where mesopredators threaten the survival of native species, reliable density estimates are important to formulate appropriate management strategies (e.g., population control vs. eradication) and evaluate efficacy of different management interventions (e.g., trapping vs. baiting vs. shooting). In this study, we assess the effect of camera‐trap placement on density estimates. Specifically, we compared maximum likelihood SEMR density estimates from three different spatial arrays of camera traps, including *on‐track grid*, *on‐track transect,* and *off‐track grid *(plus all cameras combined), to determine how the spatial arrangement of camera traps affects the precision of population density estimates. Moreover, we used the certainty in the identity and whereabouts of individuals (via GPS collars) to determine the rate of detection and non‐detection of marked foxes. The results inform future camera‐trap survey designs for mesopredators and other wildlife, and provide insight into how resighting rates of identifiable individuals affect populations density estimates using SEMR models.

## 2 MATERIALS AND METHODS

### 2.1 Study area

**Figure 1:**
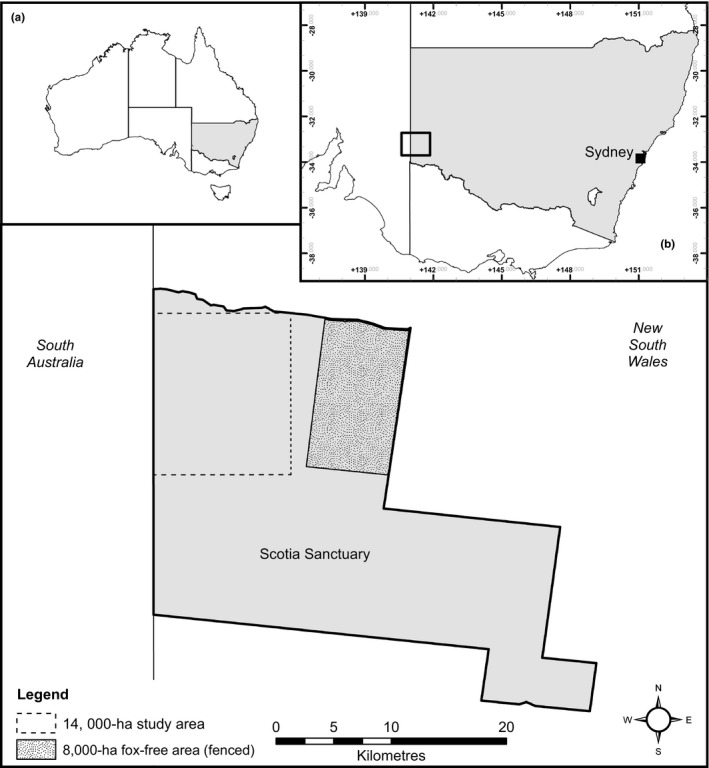
The study location within Australia (a), New South Wales (b), and Scotia Sanctuary (main figure)

Our study occurred at Scotia Sanctuary, a 64,659‐ha private conservation reserve in south‐western New South Wales, Australia (−33.15°S, 141.06°E; Figure 1) owned and managed by the Australian Wildlife Conservancy. The climate is semi‐arid with low and highly variable rainfall (spatially and temporally) that averages ~230 mm per year with high evapotranspiration (~1,500 mm/year) and low relative humidity (ave. ~20%; Australian Wildlife Conservancy, unpublished data). Cool winters (ave. max. <17°C) and hot summers (ave. max. >30°C) characterize the site, with annual temperature extremes ranging from −6 to 48°C. The landscape features stable east–west sand dunes of red sand and sandy solonized brown soil over clay (Westbrooke, Miller, & Kerr, 1998). Vegetation is dominated by three main communities: mallee *Eucalyptus *spp. open‐shrubland with a spinifex (*Triodia scariosa*) understorey, mallee open‐shrubland with a mixed‐shrub understorey (e.g., *Senna*, *Dodonaea* and *Eremophila* spp.), and *Casuarina pauper *woodland (Westbrooke et al., 1998). Red foxes are the largest predator present and their population in the study area was not subject to any form of population control during the project or in the six years prior.

### 2.2 Data collection

**Figure 2:**
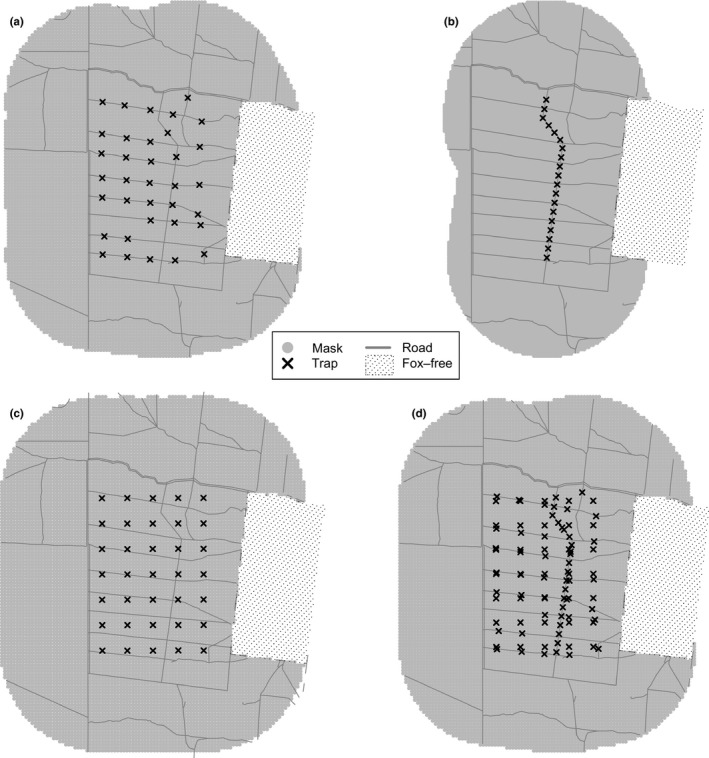
The four camera‐trap arrays used at Scotia Sanctuary, 2015–2017. (a) *on‐track grid* (35 locations); (b) *on‐track transect* (19 locations); (c) *off‐track grid* (35 locations), and (d) *all cameras* combined (98 locations, including nine supplementary cameras)

To measure fox density, we used 107 camera traps with passive‐infrared sensors (HC600; Reconyx, Holmen, WI, USA) distributed in three different “arrays” across a 14,000‐ha study area, namely *on‐track grid* (35 cameras; Figure 2a), *on‐track transect* (28 cameras; Figure 2b), and for a short period when additional resources were available, *off‐track grid* (35 cameras; Figure 2c). Nine additional cameras were placed on tracks to provide extra spatial coverage (Figure 2d), although data from these cameras were used only for analyses where all three arrays were combined (see below). Camera placement in the grid arrays was determined by dividing the study area (14 × 10 km) into 35 uniform grid cells (2 × 2 km; 400 ha). For the *on‐track grid *array, one camera was placed on a track within each grid cell, as close to the center of the cell as possible. For the *off‐track grid* array, one camera was placed at the centroid of each grid cell away from tracks. For the *on‐track transect* array, cameras were spaced at ~750‐m intervals along the length of a single track located along the approximate center of the study area. For the *on‐track transect* array, nine (of the 19) locations had paired cameras (i.e., one camera either side of the track) to provide information for a related study. Photographs of foxes recorded simultaneously by both paired cameras were recorded as one detection‐event only; hence, paired cameras had a higher detection probability than non‐paired cameras. Whether transect cameras were individual or paired was included as a covariate in the modeling process (see Section 2.4 below). The time spent in the field each month to keep cameras operational was recorded separately for each array.

We conducted 24 camera‐trapping sessions at monthly intervals for the *on‐track grid* array. The first session commenced October 1, 2015, and the final commenced September 1, 2017. Trapping sessions were the same for the *on‐track transect* array and supplementary cameras, excluding April–May 2016 (i.e., a total of 22 sessions); while three trapping sessions were conducted for the *off‐track grid* array (July, August, September 2017). Each session consisted of 24 consecutive trapping occasions (i.e., 24‐hr periods from 09:00 to 08:59 hr) unless problems were noted with camera operability, whereby trap usage was accounted for in the analysis (see Section 2.4 below).

Cameras were attached to a galvanized steel post driven into the ground, with the sensor positioned 0.5 m above ground, aimed approximately 4.5 m away “down” the center of the track (i.e., ~22° relative to the track's edge). Cameras recorded five consecutive images when triggered, with no time delay, and high image quality and trigger sensitivity. Images were stamped with camera location, date, and time. Cameras recorded monochromatic images at night and color images during the day under ambient light. No baits or lures were used at cameras.

### 2.3 Identification of individual foxes

Due to their uniform pelage, individual red foxes cannot be identified reliably from photographs unless marked artificially (Guthlin, Storch, & Kuchenhoff, 2014). To identify individuals on camera‐trap images, we fitted 28 foxes with GPS collars (Q4000E; Telemetry Solutions, Concord, CA, USA) over a three‐year period: seven foxes October 2015–March 2016, 10 foxes July–December 2016, and 11 foxes June–September 2017. Collars operated for approximately four months (before being programmed to detach from foxes automatically) and recorded location fixes at 20‐min intervals between 17:00 and 09:00 hr and at 96‐min intervals between 09:00 and 17:00 hr. Individual foxes were identified in camera‐trap images by comparing the image's time stamp with all available GPS data (additional details provided in Supporting Information Appendix S1).

### 2.4 Data analysis

Spatially explicit mark–resight (SEMR) models were fitted to the camera‐trap data using the “secr” library (v. 3.1.3; Efford, 2017a) in R (v. 3.4.3; R Core Team, 2017). In SEMR models, four pieces of information are required:

1. total number of identifiable (i.e., marked) animals available for detection in each session. Here, this was known, since the number of foxes fitted with GPS collars in the survey region was known;
2. location of traps at which animals can be detected. Here, this varied with trapping array, and varying trap usage (i.e., which cameras were operational and when) was accounted for in the analysis;
3. location at which identifiable animals were detected in each session. Here, marked foxes could move freely between camera traps and therefore be detected at multiple trap locations during each occasion. The identity of all marked foxes in photographs was determined with certainty by cross‐checking with location data from GPS collars (see Appendix S1); and
4. number of detection events of unidentified individuals at each camera‐trap location. Here, since all marked foxes were known and identifiable on photographs, all detection events of unmarked foxes were considered detection events of unidentified individuals.

We did not include the marking process in the models (i.e., the capturing of foxes to deploy GPS collars). Consequently, our data set contained some zero‐only encounter histories for foxes that were marked but never detected at any of the camera traps on any of the sampling occasions.

Four separate analyses were conducted, including (a) *on‐track grid* array only, (b) *on‐track transect* array only, (c) *off‐track grid* array only, and (d) all three arrays combined (including nine supplementary cameras).

For SEMR analyses, it is assumed that tags are not lost, which was true for GPS collars in our study. It is also assumed animal home ranges are circular and that home‐range centers are distributed in space according to a Poisson point process.

For SEMR analyses, a habitat mask is required to constrain the likelihood for computational purposes, defining a region around the trap locations beyond which the probability of detecting it is essentially zero. The mask also restricts home‐range centers to occurring in true habitat only. If activity centers are assumed to occur in non‐habitat, density estimates are biased low (i.e., animals are believed to occur within a region larger than reality). In our study, a habitat mask was created using a 4,000 m buffer around the trap locations in each survey, with inaccessible habitat removed (i.e., an 8,000‐ha fenced region that excludes foxes). The choice of a 4,000 m buffer was based on GPS location data that indicated foxes rarely moved beyond this distance.

With SEMR analyses, marked individuals are assumed to be a random sample of the larger population. Using the capture history of a marked individual, a capture function can be estimated that is conceptually consistent with a detection function in Distance Sampling approaches (Buckland et al., 2001), such that the probability of detecting an animal is assumed to be a radially declining function of the distance between an animal's (unknown) home‐range center and the (known) trap location. The capture function can take many shapes (19 capture functions are currently available within the “secr” library; v. 3.1.3; Efford, 2017a) and is typically defined by two parameters: *g*0, the probability of being trapped if the animal's home range is centered on a trap (i.e., distance between the trap and home‐range center is zero) and *σ*, a spatial scale parameter. In the current study, three null capture functions were investigated (the half‐normal, hazard‐rate, and exponential, whereby *g*0 and *σ* were constant) and the form with the lowest Akaike's Information Criterion was used thereafter (AIC, Buckland, Burnham, & Augustin, 1997). Once the form of the capture function was determined, the importance of different explanatory variables on *g*0 and *σ* was explored. Since the 24‐day trapping sessions were separated into daily intervals (occasions), we could investigate if there was a behavioral effect, whereby the capture function parameters could vary if the fox had been detected on any previous occasion during the current session (i.e., a learned response, *b*) or the occasion immediately prior (i.e., a transient response, *B*). We also investigated if detectability changed linearly with time (*T*) or with occasion (*t*). For the *on‐track transect* array, a trap‐level covariate for whether the trap had one or two cameras was investigated, and for the *all cameras *combined array, a trap‐level covariate for whether the trap was set on‐ or off‐track was investigated.

Currently, all SEMR models in “secr” are closed‐population models, so we analyzed each session separately. Estimates of fox density for each session were selected from AIC model‐averaged estimates for the nine models fitted to the data (or 10 or 11 models for *on‐track transect* and *all cameras* combined arrays, respectively).

## 3 RESULTS

Across the duration of the 24‐month study, foxes were widespread throughout the study area, being detected at all locations in the *on‐track grid* (35 trap locations), *on‐track transect* (19 trap locations), and supplementary camera (9 trap locations) arrays. During the three sessions (months) that the *off‐track grid* array was active, foxes were detected at 63% (22/35) of trap locations. When marked foxes were present for an entire session (i.e., 24‐day period), on average 26% (range 0%–100%) of those foxes were not detected by any cameras, despite their GPS data overlapping the camera arrays. Two marked foxes were never detected on camera even though they were present for 44 and 77 days (i.e., occasions), respectively. The average number of resightings of marked individuals within a session was 3 (range 0–25; for foxes present for the complete session).

**Figure 3:**
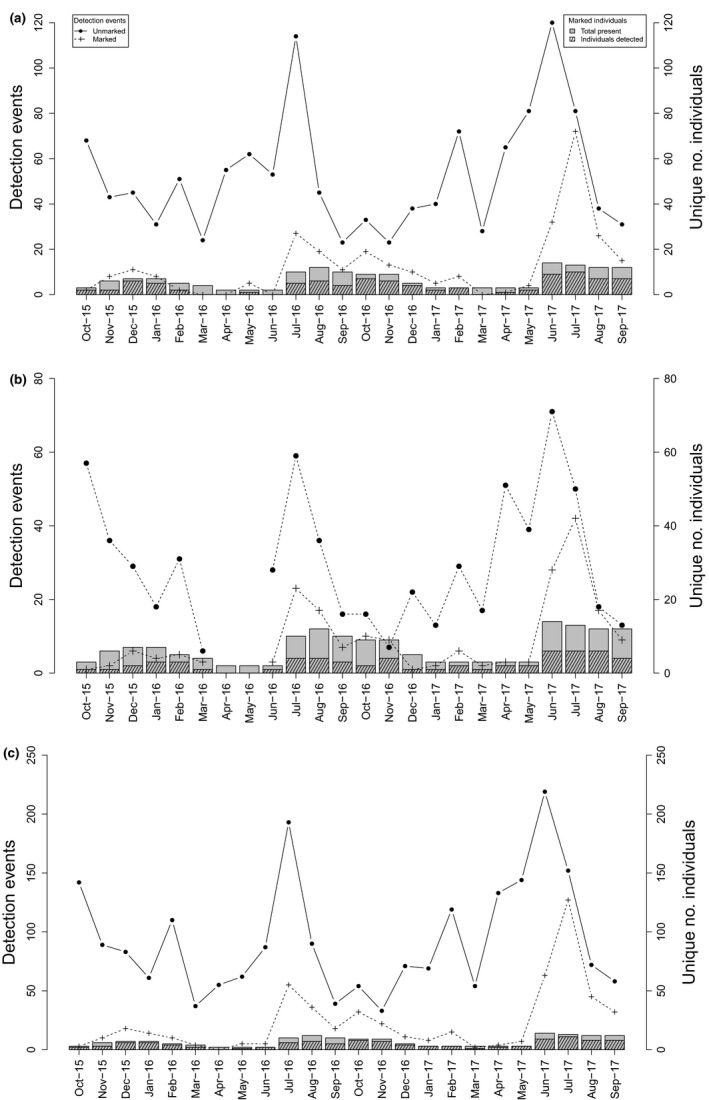
Plot of the number of detection events for marked and unmarked foxes in each session, along with the number of marked foxes present and the number of marked foxes actually detected. (a) *on‐track grid* array (24 sessions), (b) *on‐track transect *array (22 sessions), and (c) *all cameras *combined (24 sessions). See text for *off‐track grid* array (three sessions). N.B. the *y*‐axis scale varies

**Figure 4:**
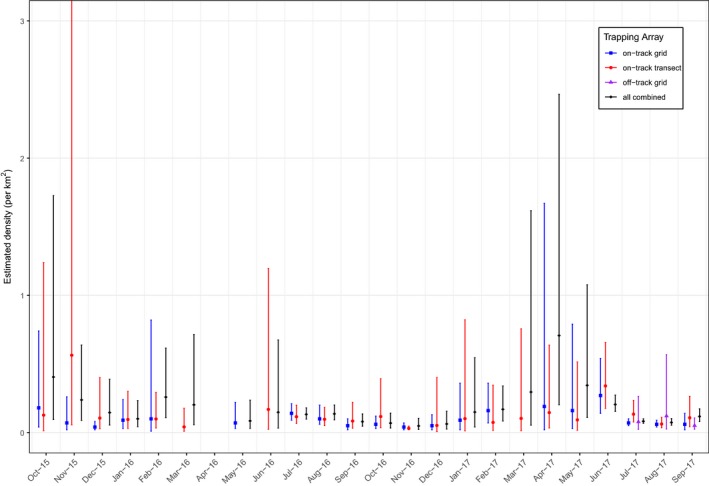
Plot of estimated fox density ±95% confidence intervals. (a) *on‐track grid* array (24 sessions trapped but estimates available for 20), (b) *on‐track transect *array (22 sessions), (c) *off‐track grid* array (three sessions), and (d) *all cameras *combined (24 sessions trapped but estimates available for 23). N.B. plot (b) November 2015 + 95% CI = 5.67

For the *on‐track grid *array, there were a total of 1,562 detection events across 24 trapping sessions, of which 1,264 and 298 were of unidentified and identified individuals, respectively. Detection events peaked for marked and unmarked foxes in July 2016 and June–July 2017 (Figure 3a, see Supporting Information Table S1 for capture information by session), coinciding with the fox mating period in south‐eastern Australia. No marked individuals were detected on cameras in March, April, June 2016 or March 2017, despite there being 4, 2, 2, and 3 marked foxes available for detection during these surveys, respectively. Consequently, density estimates using SEMR models for these sessions were not obtained (Figure 4).

For the *on‐track transect *array, there were a total of 865 detection events across 22 sessions, of which 662 and 203 were of unidentified and identified individuals, respectively. As per the *on‐track grid* array, peaks in capture events occurred in July 2016 and June–July 2017 (Figure 3b, see Supporting Information Table S2 for capture information by session). For every session, at least one marked fox was captured by one of the 19 cameras, albeit often only once or twice (i.e., October and November 2015, December 2016, January and March 2017, Supporting Information Table S2). Density was not estimated for April–May 2016 as no *on‐track transect *trapping was undertaken (Figure 4).

For the *off‐track grid *array, there were a total of 41 detection events across three sessions (July, August, September 2017), of which 28 and 13 were of unidentified and identified individuals, respectively. There were 12 or 13 marked foxes available for detection in each of the three sessions, of which only two were detected, four times only, in July 2017; two were detected, once each, in August 2017; and four were detected, a total of seven times, in September 2017. For the three sessions (July, August, September 2017), there were only 9, 11 and 8 detection events of unmarked foxes in total, respectively (see Supporting Information Table S3 for capture information by session).

Data for all three arrays (plus nine supplementary cameras) combined are presented in Figure 3c (see also, Supporting Information Table S4). In total, there were 2,773 detection events across 24 sessions (37,137 trap nights), of which 2,226 and 547 were of unidentified and identified individuals, respectively. No marked individuals were detected on cameras in April 2016, despite two marked foxes being available for detection and 840 trap nights being undertaken. Consequently, density estimates using SEMR models for that session were not obtained (Figure 4).

In all analyses, a half‐normal capture function was selected and density estimates were model averaged across all fitted models (*on‐track grid*, *off‐track grid = 9* models; *on‐track transect = 10* models; *all cameras = 11* models). Model selection output for the *all cameras* array is provided in Supporting Information Table S5. Estimates of fox density for each camera array and session are presented in Figure 4 and Supporting Information Tables S1–S4. In general, point estimates of density were similar across the trapping arrays, regardless of session (Figure 4). Where point estimates differed, there was a corresponding discrepancy in the number of marked and unmarked foxes detected between the trapping arrays.

The time spent in the field each month to keep cameras operational (i.e., maintain batteries and memory cards) varied markedly for the different camera arrays. Each month (session), an average of 2 hr 37 min was spent on the *on‐track transect *array; 10 hr 01 min on the *on‐track grid *array; 27 hr 02 min on the *off‐track grid* array; and 39 hr 41 min when *all cameras *were combined. The average distance between *off‐track grid* cameras and the nearest road was 426 m (range 30–994 m)—meaning ~30 km of off‐track walking was required each time the 35 cameras were visited. Time spent charging batteries and processing images is not presented here.

## 4 DISCUSSION

Most camera‐trap studies that generate density estimates using SECR‐based methods report on species with distinctive spots or stripes (e.g., felids) that enable individual identification from photographs (Rowcliffe et al., 2008; Wearn & Glover‐Kapfer, 2017). For species lacking uniquely identifiable pelage such as red foxes (Guthlin et al., 2014), standard SECR methods cannot be applied readily. In our study, a subset of foxes had GPS collars fitted, which enabled detection events from photographs of marked foxes to be assigned to individual animals; hence, there was no ambiguity in the identity of marked‐fox detection events. Consequently, we use this certainty in the identity and whereabouts of individuals to present an analysis based on maximum likelihood mark–resight SECR methods (i.e., SEMR), to investigate how the spatial configuration of camera‐trap surveys influence estimates of population density.

During our two‐year study, more than 100 camera traps were deployed in three spatial arrays and 28 foxes were captured and marked individually. In total, there were 2,773 detection events across 24 survey sessions and 37,137 trap nights. Despite our large survey effort, on average 26% of marked foxes were not detected in any given month even though GPS data indicated they were resident within the study area. In total, less than 20% of detection events were of marked foxes and point estimates of density were similar across the trapping arrays (Figure 4). Regardless of the trapping array, the 95% confidence intervals were always wider (i.e., uncertainty was greater) when (a) there were more detection events of unmarked foxes, (b) few marked foxes were detected, or (c) there were few resightings of marked foxes. For example, in October 2016, there were 10 detection events of two marked foxes on the *on‐track transect* array, yet on the *on‐track grid* array there was 19 detection events of seven marked foxes. This led to much narrower confidence intervals in density estimate for the *on‐track grid* array ($\hat{D}$ = 0.06 foxes per km^2^, 95% CI: [0.03, 0.12], Supporting Information Table S1) compared to the *on‐track transect* array ($\hat{D}$ = 0.11 foxes per km^2^, 95% CI: [0.03, 0.39], Supporting Information Table S2 and see Figure 4). Similarly, in October 2015, estimates from both the *on‐track grid* array and the *on‐track transect* array were very uncertain: in both instances, few marked foxes were present and there were few detections of those individuals, yet detection events of unmarked foxes were high on both arrays (68 and 57, respectively; see Figure 4, Supporting Information Tables S1 and S2).

The *all cameras* combined array typically produced density estimates with greater precision than either of the on‐track arrays individually (e.g., January, July–December 2016, June–September 2017). However, in some instances when marked foxes were not detected on either array (or by the supplementary cameras), the estimates from the *all cameras *combined array were less precise than the individual on‐track arrays. This is because, despite greater survey effort being used across the *all cameras *array, the number of detection events of marked foxes did not increase (e.g., March 2016, March 2017; Figure 4).

During July–September 2017 (winter/spring) when three different survey designs operated concurrently, the median estimated density across the three sessions was 0.06 foxes per km^2^ (CI range = 0.02–0.14) for *on‐track grid* cameras, 0.07 foxes per km^2^ (CI range = 0.02–0.56) for *off‐track grid *cameras, 0.11 foxes per km^2^ (CI range = 0.03–0.26) for *on‐track transect* cameras, and 0.08 foxes per km^2^ (CI range = 0.05–0.17) when data from *all cameras* were combined. We found that *g*0—the probability of being trapped if the animal's home range is centered on a trap—was higher when cameras were set on tracks but the magnitude of this difference was mostly small (<0.03) and varied by session (Supporting Information Table S5).

The spatial arrangement of camera traps was found to greatly influence the number of detection events of both marked and unmarked foxes, regardless of session (Figure 3), which in turn, influenced the precision of the estimates of fox density (Figure 4). In several instances, the *on‐track transect* array detected marked animals, yet in the same session, the *on‐track grid* array detected nothing (March, June 2016, and March 2017), which meant density estimates for the *on‐track grid* array could not be obtained in those sessions. Placing cameras along a single transect (e.g., road/trail), as in the *on‐track transect *array, is generally considered poor survey design because any density gradient (due to road/trail effects) cannot be estimated, since no survey effort is placed away from the transect. We found that point density estimates from the *on‐track transect* array were broadly similar to estimates from the *on‐track grid*, but mostly had greater uncertainty (Figure 4).

To examine the effect of placing cameras off‐track, for three trapping sessions when additional resources were available, we compared the *on‐track grid* array with an *off‐track grid* array. The spacing between cameras (35 in both grids) was approximately the same, but the number of detection events in the *off‐track grid* was much lower: 41 compared to 263 detections for the *on‐track grid* in the same period. In two of the three sessions (July and August 2017), the density estimates were broadly similar on both grids but the confidence limits were much wider for the *off‐track grid* (Figure 4). Conversely, in September 2017 when detection events of marked foxes on *off‐track grid *cameras increased, the confidence interval for that array was narrower than both the *on‐track grid *and *on‐track transect *arrays.

Considering only sessions in which uncertainty was lowest (i.e., July–November 2016 and July–September 2017), density estimates ranged between 0.05 and 0.14 foxes per km^2 ^when all data were combined. Comparisons with other research are difficult as there are only two published studies that use camera traps to derive density estimates for foxes in Australia. Moreover, both studies were based on substantially shorter survey periods and used different analytical approaches because no foxes were uniquely identifiable. First, Silvey, Hayward, and Gibb (2015) estimated density to be 3.0 foxes per km^2^, in the same area as the current study, using camera traps and random encounter models (Rowcliffe et al., 2008). Silvey et al. (2015) assumed a movement vector of 2.5 km per day for foxes, whereas data from 24 foxes fitted with GPS collars at Scotia show mean movement to be 14 km per day (Andrew Carter & David Roshier., unpublished data). Such a large difference in movement rates would substantially lower the density estimate of Silvey et al. (2015). This emphasizes the sensitivity of models to parametrization and the need for context‐appropriate data for reliable estimation of density.

Second, Ramsey et al. (2015) used camera traps to estimate fox density in the Grampians National Park in south‐eastern Australia using a spatially explicit presence–absence model. Like random encounter models (Rowcliffe et al., 2008), a major practical advantage of this method is that it does not require identification of individual animals. The survey area (10,000 ha) and sampling‐session duration (35 days) of Ramsey et al. (2015) were similar to the current study, and the density estimate of 0.22 foxes per km^2 ^was within the range of estimates from our overall study; albeit higher than in any of the periods identified above with low uncertainty. The density estimate of Ramsey et al. (2015) was derived from a single 35‐day sampling session, compared with our study which incorporated 24 sampling sessions over a two‐year period. When interpreting fox density estimates from short‐term studies, it should be noted that red foxes are influenced by seasonality (Marks, 2001), with activity and density varying throughout the year in response to their life cycle and breeding pattern.

For the development of an operational means to estimate density, the amount of effort to deploy and maintain cameras is a key consideration when evaluating camera‐trap survey design (De Bondi, White, Stevens, & Cooke, 2010; Silveira, Jácomo, & Diniz‐Filho, 2003; Welbourne, MacGregor, Paull, & Lindenmayer, 2015). In our study, the field time requirements for the *off‐track grid* were 2.7 times greater than for the *on‐track grid* and >10 times greater than for the *on‐track transect *array. *Off‐track grid* cameras required more time because ~30 km of off‐track walking was required each time the 35‐camera array was visited. In contrast, *on‐track grid* and *on‐track transect *cameras were placed along roads and could be accessed more efficiently.

Our research provides the first long‐term study of red fox density combining camera traps and spatially explicit density estimation methods. Our findings demonstrate the tension between deployment of unbiased designs and the practicalities of reducing uncertainty around density estimates. That is, the need to ensure that camera placement enables frequent resightings of uniquely identifiable individuals out‐weighs concerns about the magnitude of unknown biases associated with placing cameras on roads, tracks, or trails. The comprehensive track network in our study area enabled an explicit comparison of biased (*on‐track grid*, *on‐track transect*) and unbiased survey designs (*off‐track grid*), and all arrays could produce relatively precise estimates if resightings of marked foxes were sufficiently high. Variation in precision associated with different survey designs may be exacerbated further if different individuals or classes within a population have different microhabitat preferences, as has been shown in studies of large felids (e.g., Cheyne, Stark, Limin, & Macdonald, 2013; Sollmann et al., 2011; Sollmann, Linkie, Haidir, & Macdonald, 2014). This underscores the importance of incorporating information on the spatial behavior and/or preferences of the subject species prior to commencing camera‐trap surveys, to ensure camera placement maximizes exposure to the population. Our findings suggest that wherever populations exist at low densities, an appropriate survey design is that which maximizes the likelihood that uniquely identifiable individuals will be detected and resighted because the reduced uncertainty in the estimator that this delivers will likely outweigh biases associated with any particular survey design.

Presently, there is limited capacity to incorporate animal movement information obtained from telemetry into mark–resight models within a maximum likelihood framework (Efford, 2017b, 2018; Efford & Hunter, 2018). Also, all mark–resight models in “secr” are closed‐population models. In future research, we will explore open‐population models and compare how incorporating GPS movement data into mark–resight analyses influences estimates of density using both Bayesian methods (e.g., Sollmann, Gardner, Parsons et al., 2013) and by extending trapping point transects to include animal movement (Potts et al., 2012).

## AUTHOR CONTRIBUTIONS

AC and DR conceived the ideas and designed the methodology; AC collected the data; JP managed the data analysis; AC and DR led the writing of the manuscript. All authors contributed critically to the drafts and gave final approval for publication.

## Supporting information

FigureS1

## Data Availability

The data are archived at the Movebank Data Repository (https://www.datarepository.movebank.org) as “Carter and Roshier (2019) Red fox (*Vulpes vulpes*)—Scotia, NSW Australia”. https://doi.org/10.5441/001/1.72hh609t. Access to the data has been embargoed until 01/01/2020.
