# Supplementary figures and images for: Toward reliable population density estimates of partially marked populations using spatially explicit mark–resight methods
Source: Ecol Evol. 2019 Jan 24;9(4):2131–41. doi: 10.1002/ece3.4907 (PMC6392348; doi:10.1002/ece3.4907)

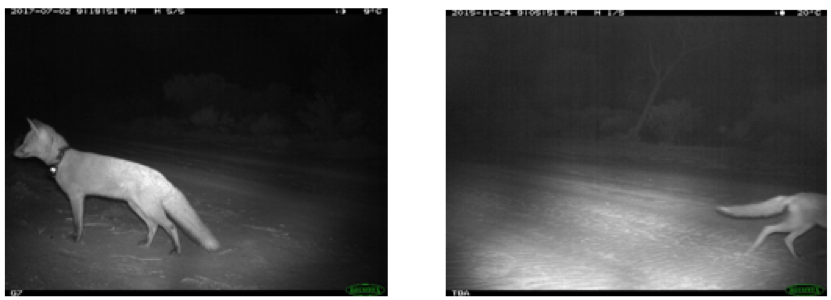

Supplement: Supplementary file 1 — FigureS1 [file ECE3-9-2131-s001.png]
