# Supplementary material for: Toward reliable population density estimates of partially marked populations using spatially explicit mark–resight methods
Source: Ecol Evol. 2019 Jan 24;9(4):2131–41. doi: 10.1002/ece3.4907 (PMC6392348; doi:10.1002/ece3.4907)
Supplement: Supplementary file 2 [file ECE3-9-2131-s002.docx]

**Supporting Information**

**Appendix S1.** Detailed methodology for identifying marked foxes from photographs.

For every fox photograph, we calculated the linear (straight-line) distance between the camera location and the location of all collared foxes at the time of the photograph using a custom Python script in ArcMAP 10.5 (Environmental Systems Research Institute Inc., Redlands, CA, USA). All analysis of fox images was conducted by the same person (A.C.), with photographs assigned to known individuals fitted with GPS collars in three difference situations:

1. “Confirmed ID”. Photograph clearly showed fox wearing a collar (Fig. S1a) and: (a) the camera was located within the fox’s known ranging area and (b), the fox’s movement path was in close proximity to the camera at the time of the photograph. If these criteria were met for >1 fox simultaneously, the photograph was assigned to the individual whose travel path was deemed most likely to encounter the camera at the time of the photograph.
2. “Presumed ID”. Photograph clearly showed fox wearing a collar but analysis of GPS data indicated it was not a fox with an active GPS collar. In three instances, GPS collars failed to drop remotely as programmed and these foxes continued to appear on cameras throughout the study period. Also, in year two and year three of the study, collars were synchronised to start recording locations simultaneously, meaning there was a lag period between fox capture and GPS collars commencing operation. In these instances, the prior- (three fox collars that did not drop) or future-ranging area of the foxes was used to determine which fox was most likely photographed at the camera in question.
3. “Inferred ID”. The neck region of the fox was obscured (Fig. S1b) but a GPS location for a collared fox was taken within 10 mins of the photograph’s time stamp and was located ≤560 metres from the camera. We deemed 10 mins to be a reasonable period of time in which to infer movement paths from GPS data and the threshold of 560 metres was derived from 113 instances of “Confirmed ID” (see above), whereby foxes travelled, on average, 56 metres per min from the time their photograph was taken until the next GPS point was recorded (hence 10 mins × 56 metres = 560 m threshold). The photograph was assigned to an individual fox if these criteria were met and a fox’s movement path was deemed likely to encounter the camera at the time of the photograph. If these criteria were met for >1 fox simultaneously, the photograph was assigned to the individual whose travel path was deemed most likely to encounter the camera at the time of the photograph.

Across the 24 survey sessions of this study, a total of 547 images were of marked foxes, including 148 Confirmed ID, 371 Presumed ID and 28 Inferred ID.

**Fig. S1.** Examples of camera-trap images showing collared foxes: (a) “Confirmed ID”, (b) “Inferred ID”, whereby the neck was obscured but subsequent analysis of GPS data indicated it was a marked fox.

**Fig. S1.**
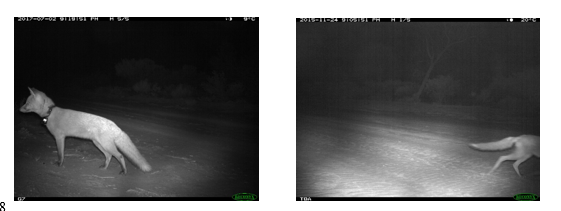


(b)

(a)

**Table S1.** Summary of capture information for the *on-track grid* array.

| **Session** | **Date** | **Trap.**  **nights*^a^*** | **No.marked.foxes** | **Uniq.**  **foxes*^b^*** | **Prop.marked.**  **dets*^c^*** | **Marked.dets*^d^*** | **Unmarked.dets*^e^*** | **Total.dets** | $\hat{\boldsymbol{D}}$***^f^*** |
| --- | --- | --- | --- | --- | --- | --- | --- | --- | --- |
| 1 | Oct-15 | 840 | 3 | 2 | 67 | 2 | 68 | 70 | 0.18(0.04,0.74) |
| 2 | Nov-15 | 840 | 6 | 2 | 33 | 8 | 43 | 51 | 0.07(0.02,0.26) |
| 3 | Dec-15 | 840 | 7 | 6 | 86 | 11 | 45 | 56 | 0.04(0.02,0.08) |
| 4 | Jan-16 | 830 | 7 | 5 | 71 | 8 | 31 | 39 | 0.09(0.03,0.24) |
| 5 | Feb-16 | 840 | 5 | 2 | 40 | 2 | 51 | 53 | 0.1(0.01,0.82) |
| 6 | Mar-16 | 840 | 4 | 0 | 0 | 0 | 24 | 24 | NA |
| 7 | Apr-16 | 840 | 2 | 0 | 0 | 0 | 55 | 55 | NA |
| 8 | May-16 | 840 | 2 | 1 | 50 | 5 | 62 | 67 | 0.07(0.03,0.22) |
| 9 | Jun-16 | 840 | 2 | 0 | 0 | 0 | 53 | 53 | NA |
| 10 | Jul-16 | 840 | 10 | 5 | 50 | 27 | 114 | 141 | 0.14(0.09,0.21) |
| 11 | Aug-16 | 840 | 12 | 6 | 50 | 19 | 45 | 64 | 0.1(0.06,0.2) |
| 12 | Sep-16 | 840 | 10 | 4 | 40 | 11 | 23 | 34 | 0.05(0.02,0.1) |
| 13 | Oct-16 | 833 | 9 | 7 | 78 | 19 | 33 | 52 | 0.06(0.03,0.12) |
| 14 | Nov-16 | 840 | 9 | 6 | 67 | 13 | 23 | 36 | 0.04(0.02,0.07) |
| 15 | Dec-16 | 840 | 5 | 4 | 80 | 10 | 38 | 48 | 0.05(0.02,0.13) |
| 16 | Jan-17 | 840 | 3 | 2 | 67 | 5 | 40 | 45 | 0.09(0.02,0.36) |
| 17 | Feb-17 | 840 | 3 | 3 | 100 | 8 | 72 | 80 | 0.16(0.07,0.36) |
| 18 | Mar-17 | 840 | 3 | 0 | 0 | 0 | 28 | 28 | NA |
| 19 | Apr-17 | 840 | 3 | 1 | 33 | 1 | 65 | 66 | 0.19(0.02,1.67) |
| 20 | May-17 | 840 | 3 | 2 | 67 | 4 | 81 | 85 | 0.16(0.03,0.79) |
| 21 | Jun-17 | 840 | 14 | 9 | 64 | 32 | 120 | 152 | 0.27(0.14,0.54) |
| 22 | Jul-17 | 840 | 13 | 10 | 77 | 72 | 81 | 153 | 0.07(0.05,0.1) |
| 23 | Aug-17 | 840 | 12 | 7 | 58 | 26 | 38 | 64 | 0.06(0.04,0.09) |
| 24 | Sep-17 | 840 | 12 | 7 | 58 | 15 | 31 | 46 | 0.06(0.02,0.14) |

*^a^*Accounting for cameras that were not operational for the duration of a session

*^b^*Number of marked foxes detected

*^c^*Proportion of marked foxes detected

*^d^*Number of detection events of marked foxes

*^e^*Number of detection events of unmarked foxes

*^f^*Estimated density (no. foxes per km^2^) plus 95% confidence intervals

**Table S2.** Summary of capture information for the *on-track transect* array.

| **Session** | **Date** | **Trap.**  **nights*^a^*** | **No.marked.foxes** | **Uniq.**  **foxes*^b^*** | **Prop.marked.**  **dets*^c^*** | **Marked.dets*^d^*** | **Unmarked.dets*^e^*** | **Total.dets** | $\hat{\boldsymbol{D}}$***^f^*** |
| --- | --- | --- | --- | --- | --- | --- | --- | --- | --- |
| 1 | Oct-15 | 431 | 3 | 1 | 33 | 1 | 57 | 58 | 0.12(0.01,1.23) |
| 2 | Nov-15 | 426 | 6 | 1 | 17 | 2 | 36 | 38 | 0.56(0.05,5.67) |
| 3 | Dec-15 | 412 | 7 | 2 | 29 | 6 | 29 | 35 | 0.10 (0.02,0.40) |
| 4 | Jan-16 | 398 | 7 | 3 | 43 | 4 | 18 | 22 | 0.09 (0.02,0.29) |
| 5 | Feb-16 | 436 | 5 | 3 | 60 | 5 | 31 | 36 | 0.09 (0.03,0.29) |
| 6 | Mar-16 | 433 | 4 | 1 | 25 | 3 | 6 | 9 | 0.04 (0.01,0.17) |
| 7 | Apr-16 | NA | 2 | NA | NA | NA | NA | NA | NA |
| 8 | May-16 | NA | 2 | NA | NA | NA | NA | NA | NA |
| 9 | Jun-16 | 456 | 2 | 1 | 50 | 3 | 28 | 31 | 0.16(0.02,1.19) |
| 10 | Jul-16 | 456 | 10 | 4 | 40 | 23 | 59 | 82 | 0.11 (0.06,0.19) |
| 11 | Aug-16 | 456 | 12 | 4 | 33 | 17 | 36 | 53 | 0.09 (0.05,0.18) |
| 12 | Sep-16 | 456 | 10 | 3 | 30 | 7 | 16 | 23 | 0.0(0.03,0.22) |
| 13 | Oct-16 | 456 | 9 | 2 | 22 | 10 | 16 | 26 | 0.11 (0.03,0.39) |
| 14 | Nov-16 | 456 | 9 | 4 | 44 | 9 | 7 | 16 | 0.03 (0.02,0.04) |
| 15 | Dec-16 | 456 | 5 | 1 | 20 | 1 | 22 | 23 | 0.05 (0.01,0.40) |
| 16 | Jan-17 | 456 | 3 | 1 | 33 | 2 | 13 | 15 | 0.10 (0.012,0.82) |
| 17 | Feb-17 | 456 | 3 | 2 | 67 | 6 | 29 | 35 | 0.07(0.015,0.34) |
| 18 | Mar-17 | 456 | 3 | 1 | 33 | 2 | 17 | 19 | 0.10(0.014,0.75) |
| 19 | Apr-17 | 456 | 3 | 2 | 67 | 3 | 51 | 54 | 0.14 (0.03,0.63) |
| 20 | May-17 | 456 | 3 | 2 | 67 | 3 | 39 | 42 | 0.09(0.02,0.51) |
| 21 | Jun-17 | 456 | 14 | 6 | 43 | 28 | 71 | 99 | 0.33 (0.17,0.65) |
| 22 | Jul-17 | 456 | 13 | 6 | 46 | 42 | 50 | 92 | 0.13 (0.076,0.23) |
| 23 | Aug-17 | 456 | 12 | 6 | 50 | 17 | 18 | 35 | 0.06 (0.03,0.11) |
| 24 | Sep-17 | 456 | 12 | 4 | 33 | 9 | 13 | 22 | 0.11 (0.04,0.26) |

*^a^*Accounting for cameras that were not operational for the duration of a session

*^b^*Number of marked foxes detected

*^c^*Proportion of marked foxes detected

*^d^*Number of detection events of marked foxes

*^e^*Number of detection events of unmarked foxes

*^f^*Estimated density (no. foxes per km^2^) plus 95% confidence intervals

**Table S3.** Summary of capture information for the *off-track grid* array.

| **Session** | **Date** | **Trap.**  **nights*^a^*** | **No.marked.foxes** | **Uniq.**  **foxes*^b^*** | **Prop.marked.**  **dets*^c^*** | **Marked.dets*^d^*** | **Unmarked.dets*^e^*** | **Total.dets** | $\hat{\boldsymbol{D}}$***^f^*** |
| --- | --- | --- | --- | --- | --- | --- | --- | --- | --- |
| 22 | Jul-17 | 792 | 13 | 2 | 15 | 4 | 9 | 13 | 0.07(0.02, 0.26) |
| 23 | Aug-17 | 798 | 12 | 2 | 17 | 2 | 11 | 13 | 0.12 (0.02,0.56) |
| 24 | Sep-17 | 840 | 12 | 4 | 33 | 7 | 8 | 15 | 0.05 (0.02,0.11) |

*^a^*Accounting for cameras that were not operational for the duration of a session

*^b^*Number of marked foxes detected

*^c^*Proportion of marked foxes detected

*^d^*Number of detection events of marked foxes

*^e^*Number of detection events of unmarked foxes

*^f^*Estimated density (no. foxes per km^2^) plus 95% confidence intervals

**Table S4.** Summary of capture information for *all cameras* combined (i.e., 35 on-track cameras, 19 on-track transect cameras, 9 supplemental cameras, and, where applicable, 35 off-track cameras).

| **Session** | **Date** | **Trap.**  **nights*^a^*** | **No.marked.foxes** | **Uniq.**  **foxes*^b^*** | **Prop.marked.**  **dets*^c^*** | **Marked.dets*^d^*** | **Unmarked.dets*^e^*** | **Total.dets** | $\hat{\boldsymbol{D}}$***^f^*** |
| --- | --- | --- | --- | --- | --- | --- | --- | --- | --- |
| 1 | Oct-15 | 1487 | 3 | 2 | 67 | 3 | 142 | 145 | 0.4(0.09,1.73) |
| 2 | Nov-15 | 1482 | 6 | 3 | 50 | 10 | 89 | 99 | 0.24(0.09,0.64) |
| 3 | Dec-15 | 1468 | 7 | 6 | 86 | 18 | 83 | 101 | 0.15(0.05,0.39) |
| 4 | Jan-16 | 1441 | 7 | 6 | 86 | 14 | 61 | 75 | 0.1(0.04,0.23) |
| 5 | Feb-16 | 1492 | 5 | 4 | 80 | 10 | 110 | 120 | 0.26(0.11,0.62) |
| 6 | Mar-16 | 1472 | 4 | 2 | 50 | 4 | 37 | 41 | 0.2(0.06,0.71) |
| 7 | Apr-16 | 840 | 2 | 0 | 0 | 0 | 55 | 55 | NA |
| 8 | May-16 | 840 | 2 | 1 | 50 | 5 | 62 | 67 | 0.08(0.03,0.24) |
| 9 | Jun-16 | 1512 | 2 | 2 | 100 | 5 | 87 | 92 | 0.15(0.03,0.67) |
| 10 | Jul-16 | 1512 | 10 | 6 | 60 | 55 | 193 | 248 | 0.13(0.1,0.18) |
| 11 | Aug-16 | 1512 | 12 | 7 | 58 | 36 | 90 | 126 | 0.14(0.09,0.2) |
| 12 | Sep-16 | 1512 | 10 | 5 | 50 | 18 | 39 | 57 | 0.08(0.05,0.14) |
| 13 | Oct-16 | 1505 | 9 | 8 | 89 | 32 | 54 | 86 | 0.07(0.03,0.14) |
| 14 | Nov-16 | 1512 | 9 | 7 | 78 | 22 | 33 | 55 | 0.05(0.02,0.1) |
| 15 | Dec-16 | 1512 | 5 | 4 | 80 | 11 | 71 | 82 | 0.06(0.03,0.16) |
| 16 | Jan-17 | 1512 | 3 | 3 | 100 | 8 | 69 | 77 | 0.15(0.04,0.55) |
| 17 | Feb-17 | 1512 | 3 | 3 | 100 | 15 | 119 | 134 | 0.17(0.08,0.34) |
| 18 | Mar-17 | 1512 | 3 | 1 | 33 | 2 | 54 | 56 | 0.3(0.05,1.62) |
| 19 | Apr-17 | 1512 | 3 | 2 | 67 | 4 | 133 | 137 | 0.71(0.2,2.47) |
| 20 | May-17 | 1512 | 3 | 3 | 100 | 7 | 144 | 151 | 0.34(0.11,1.08) |
| 21 | Jun-17 | 1512 | 14 | 9 | 64 | 63 | 219 | 282 | 0.21(0.15,0.27) |
| 22 | Jul-17 | 2304 | 13 | 12 | 92 | 128 | 152 | 280 | 0.08(0.07,0.1) |
| 23 | Aug-17 | 2310 | 12 | 8 | 62 | 45 | 72 | 117 | 0.07(0.05,0.1) |
| 24 | Sep-17 | 2352 | 12 | 8 | 62 | 32 | 58 | 90 | 0.12(0.08,0.17) |

*^a^*Accounting for cameras that were not operational for the duration of a session

*^b^*Number of marked foxes detected

*^c^*Proportion of marked foxes detected

*^d^*Number of detection events of marked foxes

*^e^*Number of detection events of unmarked foxes

*^f^*Estimated density (no. foxes per km^2^) plus 95% confidence intervals

**Table S5.** AIC model selection results for the fitted models in each session for *all cameras* combined, including estimates of *g0* for traps set on- and off-track for sessions 22–24.

| **Session 1 (Oct. 2015)** | | |  |  |  |  |  |  |
| --- | --- | --- | --- | --- | --- | --- | --- | --- |
|  | **model** | |  |  | **detectfn** | **npar** | **logLik** | **AIC** |
|  | D~1 | g0~1 | sigma~t | pID~1 | halfnormal | 28 | -437.78 | 931.56 |
|  | D~1 | g0~t | sigma~1 | pID~1 | halfnormal | 28 | -437.87 | 931.74 |
|  | D~1 | g0~T | sigma~1 | pID~1 | halfnormal | 5 | -465.27 | 940.54 |
|  | D~1 | g0~1 | sigma~T | pID~1 | halfnormal | 5 | -465.35 | 940.71 |
|  | D~1 | g0~OneTwo | sigma~1 | pID~1 | halfnormal | 5 | -470.19 | 950.39 |
|  | D~1 | g0~B | sigma~1 | pID~1 | halfnormal | 5 | -470.21 | 950.41 |
|  | D~1 | g0~1 | sigma~1 | pID~1 | halfnormal | 4 | -471.30 | 950.60 |
|  | D~1 | g0~1 | sigma~B | pID~1 | halfnormal | 5 | -470.40 | 950.80 |
|  | D~1 | g0~1 | sigma~b | pID~1 | halfnormal | 5 | -471.29 | 952.58 |
|  | D~1 | g0~b | sigma~1 | pID~1 | halfnormal | 5 | -471.29 | 952.59 |
|  |  |  |  |  |  |  |  |  |
| **Session 2 (Nov. 2015)** | | |  |  |  |  |  |  |
|  | **model** | |  |  | **detectfn** | **npar** | **logLik** | **AIC** |
|  | D~1 | g0~t | sigma~1 | pID~1 | halfnormal | 28 | -351.79 | 759.59 |
|  | D~1 | g0~1 | sigma~t | pID~1 | halfnormal | 28 | -351.80 | 759.61 |
|  | D~1 | g0~1 | sigma~b | pID~1 | halfnormal | 5 | -375.51 | 761.02 |
|  | D~1 | g0~1 | sigma~B | pID~1 | halfnormal | 5 | -375.61 | 761.22 |
|  | D~1 | g0~B | sigma~1 | pID~1 | halfnormal | 5 | -375.61 | 761.22 |
|  | D~1 | g0~b | sigma~1 | pID~1 | halfnormal | 5 | -375.63 | 761.25 |
|  | D~1 | g0~1 | sigma~1 | pID~1 | halfnormal | 4 | -376.95 | 761.90 |
|  | D~1 | g0~1 | sigma~T | pID~1 | halfnormal | 5 | -376.14 | 762.28 |
|  | D~1 | g0~T | sigma~1 | pID~1 | halfnormal | 5 | -376.33 | 762.67 |
|  | D~1 | g0~OneTwo | sigma~1 | pID~1 | halfnormal | 5 | -376.72 | 763.43 |
|  |  |  |  |  |  |  |  |  |
| **Session 3 (Dec. 2015)** | | |  |  |  |  |  |  |
|  | **model** | |  |  | **detectfn** | **npar** | **logLik** | **AIC** |
|  | D~1 | g0~T | sigma~1 | pID~1 | halfnormal | 5 | -396.94 | 803.89 |
|  | D~1 | g0~1 | sigma~T | pID~1 | halfnormal | 5 | -397.37 | 804.73 |
|  | D~1 | g0~b | sigma~1 | pID~1 | halfnormal | 5 | -398.83 | 807.67 |
|  | D~1 | g0~1 | sigma~b | pID~1 | halfnormal | 5 | -398.88 | 807.77 |
|  | D~1 | g0~t | sigma~1 | pID~1 | halfnormal | 28 | -380.94 | 817.88 |
|  | D~1 | g0~1 | sigma~1 | pID~1 | halfnormal | 4 | -405.49 | 818.99 |
|  | D~1 | g0~OneTwo | sigma~1 | pID~1 | halfnormal | 5 | -405.16 | 820.31 |
|  | D~1 | g0~B | sigma~1 | pID~1 | halfnormal | 5 | -405.19 | 820.38 |
|  | D~1 | g0~1 | sigma~t | pID~1 | halfnormal | 28 | -382.27 | 820.54 |
|  | D~1 | g0~1 | sigma~B | pID~1 | halfnormal | 5 | -405.46 | 820.92 |
|  |  |  |  |  |  |  |  |  |
| **Session 4 (Jan. 2016)** | | |  |  |  |  |  |  |
|  | **model** | |  |  | **detectfn** | **npar** | **logLik** | **AIC** |
|  | D~1 | g0~1 | sigma~t | pID~1 | halfnormal | 28 | -287.84 | 631.67 |
|  | D~1 | g0~t | sigma~1 | pID~1 | halfnormal | 28 | -287.85 | 631.70 |
|  | D~1 | g0~1 | sigma~1 | pID~1 | halfnormal | 4 | -315.67 | 639.33 |
|  | D~1 | g0~1 | sigma~b | pID~1 | halfnormal | 5 | -314.80 | 639.60 |
|  | D~1 | g0~OneTwo | sigma~1 | pID~1 | halfnormal | 5 | -315.53 | 641.06 |
|  | D~1 | g0~1 | sigma~B | pID~1 | halfnormal | 5 | -315.57 | 641.13 |
|  | D~1 | g0~T | sigma~1 | pID~1 | halfnormal | 5 | -315.58 | 641.15 |
|  | D~1 | g0~B | sigma~1 | pID~1 | halfnormal | 5 | -315.58 | 641.17 |
|  | D~1 | g0~1 | sigma~T | pID~1 | halfnormal | 5 | -315.64 | 641.28 |
|  | D~1 | g0~b | sigma~1 | pID~1 | halfnormal | 5 | -315.66 | 641.32 |

**Table S5.** Continued.

| **Session 5 (Feb. 2016)** | | |  |  |  |  |  |  |
| --- | --- | --- | --- | --- | --- | --- | --- | --- |
|  | **model** | |  |  | **detectfn** | **npar** | **logLik** | **AIC** |
|  | D~1 | g0~1 | sigma~t | pID~1 | halfnormal | 28 | -409.81 | 875.61 |
|  | D~1 | g0~t | sigma~1 | pID~1 | halfnormal | 28 | -409.97 | 875.93 |
|  | D~1 | g0~T | sigma~1 | pID~1 | halfnormal | 5 | -440.50 | 891.01 |
|  | D~1 | g0~1 | sigma~T | pID~1 | halfnormal | 5 | -440.59 | 891.18 |
|  | D~1 | g0~OneTwo | sigma~1 | pID~1 | halfnormal | 5 | -442.35 | 894.70 |
|  | D~1 | g0~1 | sigma~1 | pID~1 | halfnormal | 4 | -443.45 | 894.89 |
|  | D~1 | g0~1 | sigma~b | pID~1 | halfnormal | 5 | -443.26 | 896.53 |
|  | D~1 | g0~b | sigma~1 | pID~1 | halfnormal | 5 | -443.30 | 896.60 |
|  | D~1 | g0~1 | sigma~B | pID~1 | halfnormal | 5 | -443.40 | 896.80 |
|  | D~1 | g0~B | sigma~1 | pID~1 | halfnormal | 5 | -443.45 | 896.89 |
|  |  |  |  |  |  |  |  |  |
| **Session 6 (Mar. 2016)** | | |  |  |  |  |  |  |
|  | **model** | |  |  | **detectfn** | **npar** | **logLik** | **AIC** |
|  | D~1 | g0~1 | sigma~1 | pID~1 | halfnormal | 4 | -189.65 | 387.30 |
|  | D~1 | g0~1 | sigma~B | pID~1 | halfnormal | 5 | -189.32 | 388.65 |
|  | D~1 | g0~B | sigma~1 | pID~1 | halfnormal | 5 | -189.32 | 388.65 |
|  | D~1 | g0~T | sigma~1 | pID~1 | halfnormal | 5 | -189.43 | 388.86 |
|  | D~1 | g0~1 | sigma~T | pID~1 | halfnormal | 5 | -189.57 | 389.13 |
|  | D~1 | g0~OneTwo | sigma~1 | pID~1 | halfnormal | 5 | -189.60 | 389.21 |
|  | D~1 | g0~b | sigma~1 | pID~1 | halfnormal | 5 | -189.64 | 389.28 |
|  | D~1 | g0~1 | sigma~b | pID~1 | halfnormal | 5 | -189.65 | 389.30 |
|  | D~1 | g0~1 | sigma~t | pID~1 | halfnormal | 28 | -168.68 | 393.37 |
|  | D~1 | g0~t | sigma~1 | pID~1 | halfnormal | 28 | -168.90 | 393.81 |
|  |  |  |  |  |  |  |  |  |
| **Session 7 (Apr. 2016)** | | |  |  |  |  |  |  |
|  | No marked individuals were detected on cameras in this session | | | | | |  |  |
|  |  |  |  |  |  |  |  |  |
| **Session 8 (May 2016)** | | |  |  |  |  |  |  |
|  | **model** | |  |  | **detectfn** | **npar** | **logLik** | **AIC** |
|  | D~1 | g0~T | sigma~1 | pID~1 | halfnormal | 5 | -229.68 | 469.36 |
|  | D~1 | g0~1 | sigma~T | pID~1 | halfnormal | 5 | -229.94 | 469.87 |
|  | D~1 | g0~t | sigma~1 | pID~1 | halfnormal | 28 | -208.23 | 472.47 |
|  | D~1 | g0~1 | sigma~t | pID~1 | halfnormal | 28 | -209.02 | 474.04 |
|  | D~1 | g0~1 | sigma~1 | pID~1 | halfnormal | 4 | -234.03 | 476.05 |
|  | D~1 | g0~1 | sigma~B | pID~1 | halfnormal | 5 | -233.12 | 476.25 |
|  | D~1 | g0~B | sigma~1 | pID~1 | halfnormal | 5 | -233.12 | 476.25 |
|  | D~1 | g0~1 | sigma~b | pID~1 | halfnormal | 5 | -233.94 | 477.89 |
|  | D~1 | g0~b | sigma~1 | pID~1 | halfnormal | 5 | -233.99 | 477.98 |
|  | D~1 | g0~OneTwo | sigma~1 | pID~1 | halfnormal | 5 | -234.03 | 478.05 |
|  |  |  |  |  |  |  |  |  |
| **Session 9 (Jun. 2016)** | | |  |  |  |  |  |  |
|  | **model** | |  |  | **detectfn** | **npar** | **logLik** | **AIC** |
|  | D~1 | g0~OneTwo | sigma~1 | pID~1 | halfnormal | 5 | -348.47 | 706.95 |
|  | D~1 | g0~1 | sigma~1 | pID~1 | halfnormal | 4 | -349.79 | 707.58 |
|  | D~1 | g0~1 | sigma~B | pID~1 | halfnormal | 5 | -349.22 | 708.43 |
|  | D~1 | g0~B | sigma~1 | pID~1 | halfnormal | 5 | -349.22 | 708.43 |
|  | D~1 | g0~T | sigma~1 | pID~1 | halfnormal | 5 | -349.53 | 709.05 |
|  | D~1 | g0~1 | sigma~T | pID~1 | halfnormal | 5 | -349.53 | 709.07 |
|  | D~1 | g0~b | sigma~1 | pID~1 | halfnormal | 5 | -349.57 | 709.14 |
|  | D~1 | g0~1 | sigma~b | pID~1 | halfnormal | 5 | -349.70 | 709.41 |
|  | D~1 | g0~1 | sigma~t | pID~1 | halfnormal | 28 | -328.61 | 713.22 |
|  | D~1 | g0~t | sigma~1 | pID~1 | halfnormal | 28 | -329.34 | 714.67 |

**Table S5.** Continued.

| **Session 10 (Jul. 2016)** | | |  |  |  |  |  |  |
| --- | --- | --- | --- | --- | --- | --- | --- | --- |
|  | **model** | |  |  | **detectfn** | **npar** | **logLik** | **AIC** |
|  | D~1 | g0~t | sigma~1 | pID~1 | halfnormal | 28 | -707.63 | 1471.26 |
|  | D~1 | g0~1 | sigma~t | pID~1 | halfnormal | 28 | -711.23 | 1478.45 |
|  | D~1 | g0~B | sigma~1 | pID~1 | halfnormal | 5 | -766.95 | 1543.91 |
|  | D~1 | g0~1 | sigma~B | pID~1 | halfnormal | 5 | -771.77 | 1553.53 |
|  | D~1 | g0~1 | sigma~1 | pID~1 | halfnormal | 4 | -773.74 | 1555.48 |
|  | D~1 | g0~OneTwo | sigma~1 | pID~1 | halfnormal | 5 | -773.25 | 1556.51 |
|  | D~1 | g0~b | sigma~1 | pID~1 | halfnormal | 5 | -773.28 | 1556.56 |
|  | D~1 | g0~T | sigma~1 | pID~1 | halfnormal | 5 | -773.29 | 1556.59 |
|  | D~1 | g0~1 | sigma~b | pID~1 | halfnormal | 5 | -773.73 | 1557.47 |
|  | D~1 | g0~1 | sigma~T | pID~1 | halfnormal | 5 | -773.74 | 1557.48 |
|  |  |  |  |  |  |  |  |  |
| **Session 11 (Aug. 2016)** | | |  |  |  |  |  |  |
|  | **model** | |  |  | **detectfn** | **npar** | **logLik** | **AIC** |
|  | D~1 | g0~1 | sigma~t | pID~1 | halfnormal | 28 | -450.39 | 956.78 |
|  | D~1 | g0~t | sigma~1 | pID~1 | halfnormal | 28 | -452.76 | 961.52 |
|  | D~1 | g0~T | sigma~1 | pID~1 | halfnormal | 5 | -476.44 | 962.88 |
|  | D~1 | g0~1 | sigma~T | pID~1 | halfnormal | 5 | -476.82 | 963.64 |
|  | D~1 | g0~1 | sigma~b | pID~1 | halfnormal | 5 | -485.58 | 981.16 |
|  | D~1 | g0~b | sigma~1 | pID~1 | halfnormal | 5 | -485.64 | 981.29 |
|  | D~1 | g0~1 | sigma~1 | pID~1 | halfnormal | 4 | -486.65 | 981.31 |
|  | D~1 | g0~OneTwo | sigma~1 | pID~1 | halfnormal | 5 | -486.24 | 982.47 |
|  | D~1 | g0~B | sigma~1 | pID~1 | halfnormal | 5 | -486.57 | 983.14 |
|  | D~1 | g0~1 | sigma~B | pID~1 | halfnormal | 5 | -486.63 | 983.25 |
|  |  |  |  |  |  |  |  |  |
| **Session 12 (Sep. 2016)** | | |  |  |  |  |  |  |
|  | **model** | |  |  | **detectfn** | **npar** | **logLik** | **AIC** |
|  | D~1 | g0~1 | sigma~t | pID~1 | halfnormal | 28 | -224.68 | 505.37 |
|  | D~1 | g0~t | sigma~1 | pID~1 | halfnormal | 28 | -226.16 | 508.32 |
|  | D~1 | g0~1 | sigma~B | pID~1 | halfnormal | 5 | -250.97 | 511.95 |
|  | D~1 | g0~1 | sigma~T | pID~1 | halfnormal | 5 | -251.41 | 512.83 |
|  | D~1 | g0~1 | sigma~1 | pID~1 | halfnormal | 4 | -252.60 | 513.21 |
|  | D~1 | g0~T | sigma~1 | pID~1 | halfnormal | 5 | -251.64 | 513.29 |
|  | D~1 | g0~b | sigma~1 | pID~1 | halfnormal | 5 | -251.81 | 513.63 |
|  | D~1 | g0~B | sigma~1 | pID~1 | halfnormal | 5 | -251.84 | 513.67 |
|  | D~1 | g0~1 | sigma~b | pID~1 | halfnormal | 5 | -252.25 | 514.50 |
|  | D~1 | g0~OneTwo | sigma~1 | pID~1 | halfnormal | 5 | -252.33 | 514.67 |
|  |  |  |  |  |  |  |  |  |
| **Session 13 (Oct. 2016)** | | |  |  |  |  |  |  |
|  | **model** | |  |  | **detectfn** | **npar** | **logLik** | **AIC** |
|  | D~1 | g0~b | sigma~1 | pID~1 | halfnormal | 5 | -374.01 | 758.01 |
|  | D~1 | g0~1 | sigma~1 | pID~1 | halfnormal | 4 | -376.23 | 760.46 |
|  | D~1 | g0~1 | sigma~B | pID~1 | halfnormal | 5 | -375.55 | 761.11 |
|  | D~1 | g0~1 | sigma~b | pID~1 | halfnormal | 5 | -375.75 | 761.49 |
|  | D~1 | g0~OneTwo | sigma~1 | pID~1 | halfnormal | 5 | -375.79 | 761.57 |
|  | D~1 | g0~1 | sigma~T | pID~1 | halfnormal | 5 | -375.87 | 761.73 |
|  | D~1 | g0~B | sigma~1 | pID~1 | halfnormal | 5 | -375.96 | 761.92 |
|  | D~1 | g0~T | sigma~1 | pID~1 | halfnormal | 5 | -376.02 | 762.03 |
|  | D~1 | g0~1 | sigma~t | pID~1 | halfnormal | 28 | -355.69 | 767.38 |
|  | D~1 | g0~t | sigma~1 | pID~1 | halfnormal | 28 | -356.46 | 768.92 |

**Table S5.** Continued.

| **Session 14 (Nov. 2016)** | | |  |  |  |  |  |  |
| --- | --- | --- | --- | --- | --- | --- | --- | --- |
|  | **model** | |  |  | **detectfn** | **npar** | **logLik** | **AIC** |
|  | D~1 | g0~1 | sigma~b | pID~1 | halfnormal | 5 | -263.74 | 537.48 |
|  | D~1 | g0~b | sigma~1 | pID~1 | halfnormal | 5 | -264.13 | 538.26 |
|  | D~1 | g0~1 | sigma~B | pID~1 | halfnormal | 5 | -265.33 | 540.65 |
|  | D~1 | g0~1 | sigma~1 | pID~1 | halfnormal | 4 | -266.61 | 541.23 |
|  | D~1 | g0~T | sigma~1 | pID~1 | halfnormal | 5 | -266.07 | 542.14 |
|  | D~1 | g0~B | sigma~1 | pID~1 | halfnormal | 5 | -266.33 | 542.66 |
|  | D~1 | g0~1 | sigma~T | pID~1 | halfnormal | 5 | -266.48 | 542.95 |
|  | D~1 | g0~OneTwo | sigma~1 | pID~1 | halfnormal | 5 | -266.51 | 543.02 |
|  | D~1 | g0~1 | sigma~t | pID~1 | halfnormal | 28 | -245.99 | 547.97 |
|  | D~1 | g0~t | sigma~1 | pID~1 | halfnormal | 28 | -246.37 | 548.74 |
|  |  |  |  |  |  |  |  |  |
| **Session 15 (Dec. 2016)** | | |  |  |  |  |  |  |
|  | **model** | |  |  | **detectfn** | **npar** | **logLik** | **AIC** |
|  | D~1 | g0~1 | sigma~b | pID~1 | halfnormal | 5 | -316.21 | 642.41 |
|  | D~1 | g0~b | sigma~1 | pID~1 | halfnormal | 5 | -316.98 | 643.95 |
|  | D~1 | g0~OneTwo | sigma~1 | pID~1 | halfnormal | 5 | -317.21 | 644.41 |
|  | D~1 | g0~1 | sigma~1 | pID~1 | halfnormal | 4 | -319.34 | 646.68 |
|  | D~1 | g0~1 | sigma~B | pID~1 | halfnormal | 5 | -318.50 | 646.99 |
|  | D~1 | g0~B | sigma~1 | pID~1 | halfnormal | 5 | -318.50 | 646.99 |
|  | D~1 | g0~1 | sigma~T | pID~1 | halfnormal | 5 | -319.27 | 648.54 |
|  | D~1 | g0~T | sigma~1 | pID~1 | halfnormal | 5 | -319.30 | 648.59 |
|  | D~1 | g0~1 | sigma~t | pID~1 | halfnormal | 28 | -304.16 | 664.31 |
|  | D~1 | g0~t | sigma~1 | pID~1 | halfnormal | 28 | -304.51 | 665.02 |
|  |  |  |  |  |  |  |  |  |
| **Session 16 (Jan. 2017)** | | |  |  |  |  |  |  |
|  | **model** | |  |  | **detectfn** | **npar** | **logLik** | **AIC** |
|  | D~1 | g0~1 | sigma~b | pID~1 | halfnormal | 5 | -300.08 | 610.16 |
|  | D~1 | g0~1 | sigma~1 | pID~1 | halfnormal | 4 | -301.67 | 611.35 |
|  | D~1 | g0~b | sigma~1 | pID~1 | halfnormal | 5 | -300.89 | 611.79 |
|  | D~1 | g0~OneTwo | sigma~1 | pID~1 | halfnormal | 5 | -301.26 | 612.52 |
|  | D~1 | g0~1 | sigma~t | pID~1 | halfnormal | 28 | -278.26 | 612.53 |
|  | D~1 | g0~T | sigma~1 | pID~1 | halfnormal | 5 | -301.40 | 612.79 |
|  | D~1 | g0~1 | sigma~T | pID~1 | halfnormal | 5 | -301.47 | 612.95 |
|  | D~1 | g0~B | sigma~1 | pID~1 | halfnormal | 5 | -301.67 | 613.33 |
|  | D~1 | g0~1 | sigma~B | pID~1 | halfnormal | 5 | -301.67 | 613.34 |
|  | D~1 | g0~t | sigma~1 | pID~1 | halfnormal | 28 | -279.42 | 614.85 |
|  |  |  |  |  |  |  |  |  |
| **Session 17 (Feb. 2017)** | | |  |  |  |  |  |  |
|  | **model** | |  |  | **detectfn** | **npar** | **logLik** | **AIC** |
|  | D~1 | g0~1 | sigma~t | pID~1 | halfnormal | 28 | -443.18 | 942.36 |
|  | D~1 | g0~t | sigma~1 | pID~1 | halfnormal | 28 | -443.29 | 942.58 |
|  | D~1 | g0~1 | sigma~T | pID~1 | halfnormal | 5 | -477.94 | 965.88 |
|  | D~1 | g0~T | sigma~1 | pID~1 | halfnormal | 5 | -478.17 | 966.34 |
|  | D~1 | g0~1 | sigma~B | pID~1 | halfnormal | 5 | -480.23 | 970.47 |
|  | D~1 | g0~1 | sigma~b | pID~1 | halfnormal | 5 | -480.59 | 971.18 |
|  | D~1 | g0~B | sigma~1 | pID~1 | halfnormal | 5 | -480.69 | 971.38 |
|  | D~1 | g0~1 | sigma~1 | pID~1 | halfnormal | 4 | -482.18 | 972.36 |
|  | D~1 | g0~b | sigma~1 | pID~1 | halfnormal | 5 | -481.77 | 973.53 |
|  | D~1 | g0~OneTwo | sigma~1 | pID~1 | halfnormal | 5 | -482.18 | 974.35 |

**Table S5.** Continued.

| **Session 18 (Mar. 2017)** | | |  |  |  |  |  |  |
| --- | --- | --- | --- | --- | --- | --- | --- | --- |
|  | **model** | |  |  | **detectfn** | **npar** | **logLik** | **AIC** |
|  | D~1 | g0~1 | sigma~1 | pID~1 | halfnormal | 4 | -236.02 | 480.05 |
|  | D~1 | g0~1 | sigma~T | pID~1 | halfnormal | 5 | -235.36 | 480.71 |
|  | D~1 | g0~T | sigma~1 | pID~1 | halfnormal | 5 | -235.36 | 480.72 |
|  | D~1 | g0~t | sigma~1 | pID~1 | halfnormal | 28 | -212.86 | 481.71 |
|  | D~1 | g0~1 | sigma~t | pID~1 | halfnormal | 28 | -212.87 | 481.74 |
|  | D~1 | g0~1 | sigma~B | pID~1 | halfnormal | 5 | -235.88 | 481.76 |
|  | D~1 | g0~B | sigma~1 | pID~1 | halfnormal | 5 | -235.88 | 481.76 |
|  | D~1 | g0~1 | sigma~b | pID~1 | halfnormal | 5 | -236.02 | 482.04 |
|  | D~1 | g0~b | sigma~1 | pID~1 | halfnormal | 5 | -236.02 | 482.05 |
|  | D~1 | g0~OneTwo | sigma~1 | pID~1 | halfnormal | 5 | -236.02 | 482.05 |
|  |  |  |  |  |  |  |  |  |
| **Session 19 (Apr. 2017)** | | |  |  |  |  |  |  |
|  | **model** | |  |  | **detectfn** | **npar** | **logLik** | **AIC** |
|  | D~1 | g0~T | sigma~1 | pID~1 | halfnormal | 5 | -461.45 | 932.89 |
|  | D~1 | g0~1 | sigma~T | pID~1 | halfnormal | 5 | -461.49 | 932.99 |
|  | D~1 | g0~1 | sigma~t | pID~1 | halfnormal | 28 | -439.27 | 934.54 |
|  | D~1 | g0~t | sigma~1 | pID~1 | halfnormal | 28 | -439.28 | 934.56 |
|  | D~1 | g0~1 | sigma~b | pID~1 | halfnormal | 5 | -468.28 | 946.56 |
|  | D~1 | g0~b | sigma~1 | pID~1 | halfnormal | 5 | -468.76 | 947.52 |
|  | D~1 | g0~OneTwo | sigma~1 | pID~1 | halfnormal | 5 | -469.69 | 949.39 |
|  | D~1 | g0~1 | sigma~1 | pID~1 | halfnormal | 4 | -470.96 | 949.92 |
|  | D~1 | g0~1 | sigma~B | pID~1 | halfnormal | 5 | -470.73 | 951.46 |
|  | D~1 | g0~B | sigma~1 | pID~1 | halfnormal | 5 | -470.73 | 951.46 |
|  |  |  |  |  |  |  |  |  |
| **Session 20 (May 2017)** | | |  |  |  |  |  |  |
|  | **model** | |  |  | **detectfn** | **npar** | **logLik** | **AIC** |
|  | D~1 | g0~1 | sigma~1 | pID~1 | halfnormal | 4 | -496.34 | 1000.69 |
|  | D~1 | g0~1 | sigma~T | pID~1 | halfnormal | 5 | -496.07 | 1002.14 |
|  | D~1 | g0~T | sigma~1 | pID~1 | halfnormal | 5 | -496.12 | 1002.25 |
|  | D~1 | g0~OneTwo | sigma~1 | pID~1 | halfnormal | 5 | -496.13 | 1002.26 |
|  | D~1 | g0~B | sigma~1 | pID~1 | halfnormal | 5 | -496.33 | 1002.66 |
|  | D~1 | g0~1 | sigma~B | pID~1 | halfnormal | 5 | -496.33 | 1002.67 |
|  | D~1 | g0~t | sigma~1 | pID~1 | halfnormal | 28 | -466.32 | 988.64 |
|  | D~1 | g0~1 | sigma~t | pID~1 | halfnormal | 28 | -466.75 | 989.51 |
|  | D~1 | g0~1 | sigma~b | pID~1 | halfnormal | 5 | -492.75 | 995.51 |
|  | D~1 | g0~b | sigma~1 | pID~1 | halfnormal | 5 | -494.42 | 998.85 |
|  |  |  |  |  |  |  |  |  |
| **Session 21 (Jun. 2017)** | | |  |  |  |  |  |  |
|  | **model** | |  |  | **detectfn** | **npar** | **logLik** | **AIC** |
|  | D~1 | g0~1 | sigma~t | pID~1 | halfnormal | 28 | -813.07 | 1682.15 |
|  | D~1 | g0~t | sigma~1 | pID~1 | halfnormal | 28 | -815.92 | 1687.85 |
|  | D~1 | g0~b | sigma~1 | pID~1 | halfnormal | 5 | -845.07 | 1700.15 |
|  | D~1 | g0~1 | sigma~b | pID~1 | halfnormal | 5 | -847.78 | 1705.56 |
|  | D~1 | g0~1 | sigma~1 | pID~1 | halfnormal | 4 | -850.50 | 1709.00 |
|  | D~1 | g0~1 | sigma~T | pID~1 | halfnormal | 5 | -849.93 | 1709.87 |
|  | D~1 | g0~T | sigma~1 | pID~1 | halfnormal | 5 | -850.12 | 1710.25 |
|  | D~1 | g0~1 | sigma~B | pID~1 | halfnormal | 5 | -850.18 | 1710.35 |
|  | D~1 | g0~B | sigma~1 | pID~1 | halfnormal | 5 | -850.30 | 1710.61 |
|  | D~1 | g0~OneTwo | sigma~1 | pID~1 | halfnormal | 5 | -850.48 | 1710.97 |

**Table S5.** Continued.

| **Session 22 (Jul. 2017)** | | |  |  |  |  |  |  |
| --- | --- | --- | --- | --- | --- | --- | --- | --- |
|  | **model** | |  |  | **detectfn** | **npar** | **logLik** | **AIC** |
|  | D~1 | g0~onoff**^^^** | sigma~1 | pID~1 | halfnormal | 5 | -1013.36 | 2036.72 |
|  | D~1 | g0~1 | sigma~t | pID~1 | halfnormal | 28 | -1039.87 | 2135.74 |
|  | D~1 | g0~t | sigma~1 | pID~1 | halfnormal | 28 | -1045.54 | 2147.08 |
|  | D~1 | g0~1 | sigma~b | pID~1 | halfnormal | 5 | -1080.68 | 2171.35 |
|  | D~1 | g0~1 | sigma~B | pID~1 | halfnormal | 5 | -1082.04 | 2174.08 |
|  | D~1 | g0~B | sigma~1 | pID~1 | halfnormal | 5 | -1083.15 | 2176.30 |
|  | D~1 | g0~1 | sigma~T | pID~1 | halfnormal | 5 | -1084.52 | 2179.04 |
|  | D~1 | g0~1 | sigma~1 | pID~1 | halfnormal | 4 | -1085.72 | 2179.45 |
|  | D~1 | g0~T | sigma~1 | pID~1 | halfnormal | 5 | -1084.76 | 2179.53 |
|  | D~1 | g0~OneTwo | sigma~1 | pID~1 | halfnormal | 5 | -1085.38 | 2180.75 |
|  | D~1 | g0~b | sigma~1 | pID~1 | halfnormal | 5 | -1085.45 | 2180.89 |
|  | **^^^***g0* for on-track = 0.15; *g0* for off-track = 0.01 | | | | |  |  |  |
|  |  |  |  |  |  |  |  |  |
| **Session 23 (Aug. 2017)** | | |  |  |  |  |  |  |
|  | **model** | |  |  | **detectfn** | **npar** | **logLik** | **AIC** |
|  | D~1 | g0~onoff**^^^** | sigma~1 | pID~1 | halfnormal | 5 | -531.28 | 1072.55 |
|  | D~1 | g0~1 | sigma~B | pID~1 | halfnormal | 5 | -540.38 | 1090.76 |
|  | D~1 | g0~1 | sigma~T | pID~1 | halfnormal | 5 | -540.40 | 1090.80 |
|  | D~1 | g0~B | sigma~1 | pID~1 | halfnormal | 5 | -543.88 | 1097.76 |
|  | D~1 | g0~1 | sigma~t | pID~1 | halfnormal | 28 | -521.41 | 1098.81 |
|  | D~1 | g0~1 | sigma~b | pID~1 | halfnormal | 5 | -544.43 | 1098.85 |
|  | D~1 | g0~T | sigma~1 | pID~1 | halfnormal | 5 | -544.65 | 1099.31 |
|  | D~1 | g0~1 | sigma~1 | pID~1 | halfnormal | 4 | -546.87 | 1101.73 |
|  | D~1 | g0~b | sigma~1 | pID~1 | halfnormal | 5 | -546.13 | 1102.27 |
|  | D~1 | g0~OneTwo | sigma~1 | pID~1 | halfnormal | 5 | -546.21 | 1102.43 |
|  | D~1 | g0~t | sigma~1 | pID~1 | halfnormal | 28 | -525.35 | 1106.71 |
|  | **^^^***g0* for on-track = 0.03; *g0* for off-track = 0.01 | | | | |  |  |  |
|  |  |  |  |  |  |  |  |  |
| **Session 24 (Sep. 2017)** | | |  |  |  |  |  |  |
|  | **model** | |  |  | **detectfn** | **npar** | **logLik** | **AIC** |
|  | D~1 | g0~onoff**^^^** | sigma~1 | pID~1 | halfnormal | 5 | -423.93 | 857.87 |
|  | D~1 | g0~b | sigma~1 | pID~1 | halfnormal | 5 | -429.92 | 869.85 |
|  | D~1 | g0~1 | sigma~b | pID~1 | halfnormal | 5 | -430.09 | 870.18 |
|  | D~1 | g0~1 | sigma~1 | pID~1 | halfnormal | 4 | -431.32 | 870.64 |
|  | D~1 | g0~OneTwo | sigma~1 | pID~1 | halfnormal | 5 | -430.90 | 871.80 |
|  | D~1 | g0~1 | sigma~B | pID~1 | halfnormal | 5 | -431.24 | 872.48 |
|  | D~1 | g0~T | sigma~1 | pID~1 | halfnormal | 5 | -431.28 | 872.56 |
|  | D~1 | g0~B | sigma~1 | pID~1 | halfnormal | 5 | -431.29 | 872.58 |
|  | D~1 | g0~1 | sigma~T | pID~1 | halfnormal | 5 | -431.32 | 872.64 |
|  | D~1 | g0~t | sigma~1 | pID~1 | halfnormal | 28 | -412.34 | 880.68 |
|  | D~1 | g0~1 | sigma~t | pID~1 | halfnormal | 28 | -414.06 | 884.12 |
|  | **^^^***g0* for on-track = 0.04; *g0* for off-track = 0.01 | | | | |  |  |  |
